# Advanced Cellulose-Based Materials: From Nanoparticles to Complex Structures and Composites

**DOI:** 10.3390/polym18060701

**Published:** 2026-03-13

**Authors:** Selestina Gorgieva

**Affiliations:** Faculty of Mechanical Engineering, University of Maribor, Smetanova Ulica 17, 2000 Maribor, Slovenia; selestina.gorgieva@um.si

Cellulose has long been recognised as the fundamental structural component of plant cell walls and is currently established as a highly adaptable molecular scaffold for next-generation sustainable materials. Its abundance, renewability, and intrinsic physicochemical properties position cellulose at the centre of the emerging bio-based and circular materials economy. With parallel advances in nanotechnology, biotechnology, and materials processing, cellulose can now be transformed into nanoscale building blocks, namely cellulose nanocrystals (CNCs) and cellulose nanofibrils (CNFs), that exhibit high tensile strength, low density, large specific surface areas, and high aspect ratios, being exceptionally attractive for applications in high-performance composites, biomedical engineering, energy-storage and energy-conversion devices, optical materials, and sustainable alternatives to conventional plastics and leather.

Despite this rapid progress, several scientific and technological challenges continue to limit broader industrial deployment of cellulose-derived materials. One of the most significant obstacles is the scalable and energy-efficient top-down disintegration of biomass into nanoscale form. Established isolation methods, such as high-pressure homogenisation, micro grinding, and chemical pretreatments remain energy-intensive or rely on environmentally demanding reagents. Conversely, bottom-up biosynthetic routes, particularly bacterial cellulose production, offer advantages in structural purity, crystallinity, and nanoscale uniformity but require further optimisation to achieve economically viable large-scale output. Interfacial incompatibility between hydrophilic nanocellulose and hydrophobic polymer matrices presents another major challenge, limiting dispersion, reinforcing efficiency, and mechanical performance in composite systems. Addressing this issue requires advanced surface-functionalisation strategies that enhance interfacial adhesion while preserving nanocellulose integrity. A further challenge is translating nanoscale material properties into predictable macroscopic performance, which demands precise control over hierarchical assembly from the nanometre to the bulk scale. To address these challenges and to highlight emerging strategies in this rapidly evolving field, this Special Issue entitled “Advanced Cellulose-Based Materials: From Nanoparticles to Complex Structures and Composites” was organised. The collected contributions demonstrate the breadth of ongoing research, ranging from nanocellulose processing and functionalisation to the development of porous materials, bio-based composites, and engineered nano-macro, 3D and 4D architectures.

A relevant research direction highlighted in this Special Issue concerns cellulose-based porous materials for acoustic insulation applications. Seciureanu et al. (contribution 1) investigated foam-formed porous materials derived from cellulose fibers, demonstrating their potential as sustainable alternatives to conventional synthetic acoustic insulation materials. Their results revealed that the internal pore structure and distribution significantly influence sound absorption and transmission loss, emphasising the importance of controlling the porous architecture in the design of cellulose-based acoustic materials. Building upon this concept, Nastac et al. (contribution 2) explored the modification of cellulose foams using hemicellulose-derived xylan derivatives to enhance structural integrity and surface properties. Their study demonstrated that introducing xylan-based surface modifiers significantly reduces the wettability of cellulose foams while improving mechanical stability, without compromising acoustic performance. This work highlights the potential of integrating various plant-derived polysaccharides to develop multifunctional, fully bio-based material systems. Another prominent theme concerns the development of nanocellulose-reinforced composite materials. Hwang et al. (contribution 3) investigated the enzymatic pretreatment of cellulose nanofibrils followed by spray drying to produce nanofibril powders suitable for composite processing. Their findings demonstrate that enzyme-assisted processing can significantly reduce energy consumption during nanofibril production while maintaining the reinforcing capabilities of the resulting nanocellulose in polypropylene composites. Such approaches represent an important step toward more sustainable and scalable nanocellulose manufacturing technologies. Advances in cellulose-based materials for energy-related applications are represented in work of Hren et al. (contribution 4) reporting the development of fully polysaccharide-based anion exchange membranes composed of chitosan reinforced with neat or quaternized cellulose nanofibrils. These membranes exhibited significantly improved mechanical properties, ionic conductivity, and power density when tested in direct ethanol fuel cells, demonstrating the potential of cellulose nanomaterials as structural and functional components in electrochemical energy systems. In addition to nanocomposites and membranes, several contributions explore the engineering of hierarchical cellulose architectures. Wang et al. (contribution 5) reported the fabrication of compressible and elastic cellulose wood structures using deep eutectic solvents combined with alkaline treatment. Selective removal of lignin and hemicellulose produced a honeycomb-like cellular architecture capable of reversible compression and high elasticity. Such structures may enable new applications in sensing technologies, filtration systems, and biomedical scaffolds.

The application of nanotechnology to traditional cellulose-based industries is addressed in work of Maślana et al. (contribution 6). In this paper, the strategies to enhance the retention of inorganic fillers during papermaking by using nanoparticle additives, including silica nanospheres, titanium dioxide nanoparticles, and boron nitride nanoflakes were investigated. Their study demonstrated that silica nanoparticles significantly enhance calcium carbonate retention, offering new opportunities to improve paper performance and production efficiency. The versatility of cellulose-based materials is further illustrated by the work of Bautista-Morenilla et al. (contribution 7), who investigated microblasting with powdered cellulose as a dry-cleaning technique for paper-based artworks. This approach proved effective for removing contaminants from printed materials without causing morphological damage to the paper substrate, providing a sustainable and compatible method for cultural heritage conservation. In a comprehensive review Potočnik et al. (contribution 8) summarise recent progress in the biosynthesis and modification of bacterial cellulose using synthetic biology approaches. The authors highlight advances in the genetic and metabolic engineering of cellulose-producing bacteria and discuss the potential to design microbial systems capable of producing cellulose with tailored structural and functional properties. Beyond the studies presented in here, the bacterial cellulose has emerged as a particularly promising platform for the development of advanced materials, from applications in wound dressings, drug delivery systems, and tissue engineering scaffolds [1,2,3,4]. Furthermore, the incorporation of conductive polymers or nanoparticles into bacterial cellulose matrices has enabled the development of flexible electronic devices, biosensors, and energy storage systems [5,6,7].

Collectively, the research contributions in this Special Issue demonstrate substantial progress in the development of cellulose-based materials. Nevertheless, several challenges and emerging opportunities will shape the field’s future trajectory. Sustainable and scalable nanocellulose production remains central, with greener processing routes integrating enzymatic pretreatment, mechanical fibrillation, and solvent-based fractionation gaining attention for reducing energy consumption and chemical waste. Advanced surface-functionalisation strategies, including click chemistry, bioinspired coatings, and enzymatic grafting, will broaden the functional spectrum and improve compatibility with diverse material systems. At the structural level, mastering supramolecular interactions and hierarchical organisation remains crucial for connecting nanoscale attributes with macroscopic properties. Emerging fabrication methods such as directed self-assembly, templating, and additive manufacturing are expected to provide new levels of structural control.

Future cellulose-based systems will increasingly integrate multifunctionality, combining mechanical strength with electrical conductivity, catalytic activity, or environmental responsiveness. Finally, although cellulose is inherently renewable and biodegradable, the environmental impacts of specific processing routes must be carefully evaluated. Integrating life-cycle assessment, green-chemistry principles, and recyclable process design will be essential to ensure that cellulose-based materials meaningfully contribute to global sustainability goals. With rapid progress in nanocellulose engineering, hierarchical structuring, and synthetic-biology-driven biofabrication, cellulose is poised to remain a foundation for advanced sustainable materials.
